# Motor performance in five-year-old extracorporeal membrane oxygenation survivors: a population-based study

**DOI:** 10.1186/cc7770

**Published:** 2009-04-02

**Authors:** Maria WG Nijhuis-van der Sanden, Monique HM van der Cammen-van Zijp, Anjo JWM Janssen, Jolanda JCM Reuser, Petra Mazer, Arno FJ van Heijst, Saskia J Gischler, Dick Tibboel, Louis AA Kollée

**Affiliations:** 1 Department of Paediatric Physical Therapy and Scientific Institute for Quality of Healthcare, Radboud University Nijmegen Medical Centre, Geert Grooteplein-Zuid 10, 6525 GA Nijmegen, The Netherlands; 2 Department of Physical Therapy, Pediatric Physical Therapy, Sophia Children's Hospital, Erasmus MC – University Medical Center Rotterdam, The Netherlands; 3 Department of Medical Psychology, Radboud University Nijmegen Medical Centre, Geert Grooteplein-Zuid 10, 6525 GA Nijmegen, The Netherlands; 4 Department of Pediatric Surgery, Sophia Children's Hospital, Erasmus MC – University Medical Center Rotterdam, The Netherlands; 5 Department of Neonatology, Radboud University Nijmegen Medical Centre, Geert Grooteplein-Zuid 10, 6525 GA Nijmegen, The Netherlands

## Abstract

**Introduction:**

Veno-arterial extracorporeal membrane oxygenation (VA-ECMO) is a cardio-pulmonary bypass technique to provide life support in acute reversible cardio-respiratory failure when conventional management is not successful. Most neonates receiving ECMO suffer from meconium aspiration syndrome (MAS), congenital diaphragmatic hernia (CDH), sepsis or persistent pulmonary hypertension (PPH). In five-year-old children who underwent VA-ECMO therapy as neonates, we assessed motor performance related to growth, intelligence and behaviour, and the association with the primary diagnosis.

**Methods:**

In a prospective population-based study (n = 224) 174 five-year-old survivors born between 1993 and 2000 and treated in the two designated ECMO centres in the Netherlands (Radboud University Medical Centre Nijmegen and Sophia Children's Hospital, Erasmus MC – University Medical Center Rotterdam) were invited to undergo follow-up assessment including a paediatric assessment, the movement assessment battery for children (MABC), the revised Amsterdam intelligence test (RAKIT) and the child behaviour checklist (CBCL).

**Results:**

Twenty-two percent of the children died before the age of five, 86% (n = 149) of the survivors were assessed. Normal development in all domains was found in 49% of children. Severe disabilities were present in 13%, and another 9% had impaired motor development combined with cognitive and/or behavioural problems. Chi-squared tests showed adverse outcome in MABC scores (*P *< 0.001) compared with the reference population in children with CDH, sepsis and PPH, but not in children with MAS. Compared with the Dutch population height, body mass index (BMI) and weight for height were lower in the CDH group (*P *< 0.001). RAKIT and CBCL scores did not differ from the reference population. Total MABC scores, socio-economic status, growth and CBCL scores were not related to each other, but negative motor outcome was related to lower intelligence quotient (IQ) scores (r = 0.48, *P *< 0.001).

**Conclusions:**

The ECMO population is highly at risk for developmental problems, most prominently in the motor domain. Adverse outcome differs between the primary diagnosis groups. Objective evaluation of long-term developmental problems associated with this highly invasive technology is necessary to determine best evidence-based practice. The ideal follow-up programme requires an interdisciplinary team, the use of normal-referenced tests and an international consensus on timing and actual outcome measurements.

## Introduction

Extracorporeal membrane oxygenation (ECMO) is an effective treatment for respiratory failure in neonates suffering from meconium aspiration syndrome (MAS), congenital diaphragmatic hernia (CDH), sepsis or persistent pulmonary hypertension (PPH).

ECMO is associated with high survival rates (76%) [1,2]. Nevertheless, survivors may suffer from long-term morbidity such as pulmonary dysfunction and cerebral damage, depending on the severity of primary illness and respiratory failure prior to ECMO and several factors during ECMO [3-5]. Prediction of long-term outcome after ECMO is not easy. Although neonatal brain injury tends to affect neuropsychological status at five years of age, evidence of functional recovery following brain injury has also been found [6,7].

Most studies in ECMO survivors have so far focused on the health status at the age of one to three years [1,3,8-10]. Percentages of abnormal outcome in these studies differ, probably as a result of differences in study populations, assessment procedures and inclusion criteria. The range of morbidity widens after the first year of life when more precise assessment of cognition, coordination and behaviour is feasible. Long-term longitudinal follow-up would therefore seem essential for evaluating ECMO results [11,12]. Only a few studies have focused on neuromotor outcome from the age of four years and none of these analysed the relation between motor performance and health condition, cognition and behaviour [12-14].

Previously, we presented the overall morbidity in Dutch ECMO survivors at the age of five years [15]. Six of the 98 children had major disabilities and 24 of the remaining 92 (26%) showed motor problems. The present study aims to evaluate in detail the characteristics of motor performance at five years of age in a larger Dutch ECMO population. We hypothesised that the motor performance profile is associated with the primary diagnosis. Moreover, we analysed the relations between motor performance problems, health status, cognitive and behavioural problems.

## Materials and methods

### Patients

**Figure 1:**
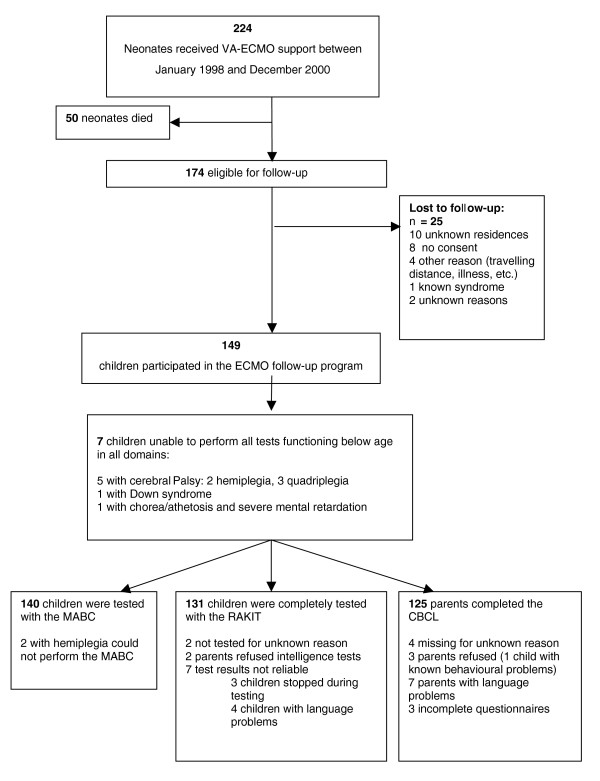
Flowchart of children included in the VA-ECMO follow-up programme at the age of five years. CBCL = child behaviour checklist; MABC = movement assessment battery for children; RAKIT = revised Amsterdam intelligence test; VA-ECMO = veno-arterial extracorporeal membrane oxygenation.

A population who had received veno-arterial (VA)-ECMO support within 28 days of birth in either of the two ECMO centres in the Netherlands (Radboud University Medical Centre Nijmegen and Sophia Children's Hospital, Erasmus MC – University Medical Center Rotterdam) between January 1993 (Nijmegen) or 1996 (Rotterdam) and December 2000 (n = 224) and were alive at age five years (n = 174) were invited to undergo follow-up assessment (Figure 1). Neonatal data had been prospectively collected in an ECMO database.

According to a national consensus on neonatal follow-up and the obligation to provide these data based on reports of the Dutch Ministry of Health, the assessment protocol is the Dutch standard of care in ECMO follow-up [16,17]. As a consequence no written informed consent from the parents was necessary. All parents were routinely informed about the long-term follow-up programme and use of anonymous data for study proposal in the neonatal period of life and again when they were invited for the assessments at the age of five years. The ethical review boards in both Medical Centres were informed and agreed with the study.

### Assessment protocol

Perinatal characteristics (Table 1) were recorded as submitted to the Extracorporeal Life Support Organization registry [2]. A paediatrician, a paediatric physical therapist and a child psychologist assessed the five-year-olds during a single clinic visit.

**Table 1:** Perinatal and extracorporeal membrane oxygenation (ECMO) characteristics of all survivors (N = 174)

	Infants not participating (n = 25)	Infants in follow-up (n = 149)
Male/Female^1^: number	17/8	87/62
Birth weight (g)^2^: mean (SD)	3272 (560)	3367 (604)
Gestational age (weeks)^2^: mean (SD)	38.6 (2.3)	39.5 (2.0)

Primary diagnoses^1^**:**	number	number
Meconium aspiration syndrome	13	75
Congenital diaphragmatic hernia	4	32
Sepsis	4	16
Persistent pulmonary hypertension	2	25
Pulmonary hypoplasia	2	1

Duration of the ECMO (hours)^2^: means (SD)	155 (57)	163 (64)

Neurological complications^1^: number	20 (5 missing)	120 (29 missing)
None	14	71
Haemorrhage	3	9
Cerebral infarction	0	6
Clinical epileptic insults	3	6
EEG epileptic insults	0	28

No differences were seen in perinatal characteristics between infants not participating and infants in follow-up;

^1 ^Chi-squared test: *P *> 0.05; ^2 ^Student t-test *P *> 0.05

EEG = electroencephalography; SD = standard deviation.

#### Questionnaires

Parents completed a questionnaire evaluating the child's health status and employment and education of both parents. Three socio-economic classes were defined using the standard Dutch profession classification [18].

#### Paediatrician's assessment

The paediatrician performed a physical examination and took a medical history. Growth parameters were expressed in standard deviation (SD) scores using the Dutch Growth Analyser, version 3.0 (Dutch Growth Foundation, Rotterdam, the Netherlands).

#### Motor performance assessment

A paediatric physical therapist administered the movement assessment battery for children (MABC). It assesses skills related to motor functioning in daily life. The MABC has four age bands each with eight items grouped into three performance sections: manual dexterity (three items), ball skills (two items) and static and dynamic balance (three items). All items are scored quantitatively (duration in seconds or number of hits or errors). Section scores for manual dexterity, ball skills and static and dynamic balance, and a total impairment score can be calculated, with lower scores indicating better performance. Scores can be interpreted using the percentile normal data tables in the manual [19,20]. Scores above the 15th percentile are considered 'normal', between 5th and 15th percentile is considered 'borderline', and below the 5th percentile is 'definitively delayed'. A Dutch version of the test is available: interrater reliability ranges from 0.70 to 0.89, while test–retest reliability is 0.75 [19,20]. The test is the most frequently used test to identify children with functional motor problems [19,21,22].

#### Cognitive assessment and behaviour

A psychologist (assistant) assessed cognitive development with the short version of the revised Amsterdam intelligence test (RAKIT) – a reliable, validated, normal-referenced Dutch instrument containing six subtests [23]. Raw subtest scores are converted into standardised scores, which are then transformed into a short RAKIT intelligence quotient (IQ) with a mean of 100 and a SD of 15. 'Mild cognitive delay' was defined by a test result between -1 SD and -2 SD (IQ ≥ 70 and < 85), and 'definitive delay' by a test result lower than -2 SD (IQ < 70).

Behavioural outcome was assessed using the Dutch version of the child behaviour checklist (CBCL) for children aged 4 to 18 years [24]. The CBCL is a validated parental questionnaire and rates 113 problem behaviour items on a three-point scale (0 = not true, 1 = somewhat true, 2 = very true). The sum of all item scores results in a total score, which is recalculated into a percentile score. Scores of 59 or less are classified as 'within normal range', scores 60 to 63 as 'borderline' and scores 64 and above 'within clinical range'.

### Data analysis

Independent t-tests or chi-squared tests were used to test differences between the participating and non-participating children and to test whether the ECMO population differed significantly from the reference population in growth, motor performance, intelligence and behaviour. Two-sample t-tests and Mann-Whitney-U tests were used to examine the differences between diagnosis subgroups. Spearman correlations between motor performance scores and growth data, intelligence scores, behavioural scores and socio-economic status were calculated. *P *values less than 0.05 were considered statistically significant. All analyses were performed using SPSS version 16.

## Results

As 25 of the 174 eligible children did not participate for various reasons (Figure 1), 149 (86%) children underwent follow-up assessment. The participating and non-participating groups did not differ in perinatal and ECMO characteristics (Table 1).

Patient characteristics at age five years are presented in Table 2. Mean and SD of height, SD of body mass index and SD of weight-for-height scores for the total sample were significantly lower than those for the Dutch reference population. Post-hoc analysis revealed that growth was within the normal range in the groups of children with sepsis and persistent pulmonary hypertension (PPH). In the group of children with meconium aspiration syndrome (MAS) only SD of height was lower than the normal range (*P *< 0.03), but in the congenital diaphragmatic hernia (CDH) population all three parameters were significantly lower (*P *< 0.001): 23 children with CDH (72%) had a weight for height lower than normal (50% < -1 SD, and 22% < -2 SD). Visual problems were rare (n = 8) and previously undetected in only one child. Hearing problems were also rare (n = 8) and previously undetected in three children.

**Table 2:** Basic characteristics of the assessed group at five years of age (n= 149)

Total group (n = 149)	
	mean (SD)
Age in months	62 (2.5)
Height score *	-0.4 (1.1)
Weight for height score **	-0.3 (1.4)
BMI score ***	-0.3 (1.4)
Motor performance percentile score (n = 140)	35.6 (28.4)
Intelligence score (n = 131)	99.7 (18.1)
Behavioural score (n = 125)	50.2 (9.9)

Socio-economic status	number (%)
- low	28 (16)
- middle	76 (44)
- high	35 (20)
- unknown	35 (20)

Vision	number
- normal	121
- abnormal/no glasses^1^	2
- adequate correction with glasses	6
- abnormal with glasses	0
- unknown	20

Sense of hearing	number
- normal	123
- abnormal/no hearing aid^1^	4
- adequate correction with hearing aid	3
- abnormal with hearing aid	1
- unknown	18

*One sample t-test: *P *< 0.001; ** *P *< 0.05; *** *P *< 0.02

^1^in one child with severe cerebral palsy correction was not possible

BMI = body mass index; SD = standard deviation.

### Motor performance

Seven children were unable to partake in the assessments (Figure 1). Another two children with hemiparesis were not able to perform the MABC, so 140 children were tested with the MABC. Chi-squared tests revealed more motor problems in these children than in the reference population (*P *< 0.001); 94 children (67% vs. 85% expected) scored within the normal range, 23 children (16.5% vs. 10% expected) were classified as 'borderline' and another 23 children (16.5% vs. 5% expected) as 'definitively delayed'. Manual dexterity scores did not differ from those for the reference population. The majority of children (86%) were right-handed, as in the reference population. Proportions of children with ball skill problems were larger than in the reference population (*P *< 0.001): 82 normal (58% vs. 85% expected), 36 borderline (26% vs. 10% expected) and 22 definitively delayed (16% vs. 5% expected). The same held true for balance skills (*P *< 0.001): 93 normal (66.5% vs. 85% expected), 24 borderline (17% vs. 10% expected) and 23 definitively delayed (16.5% vs. 5% expected).

**Figure 2:**
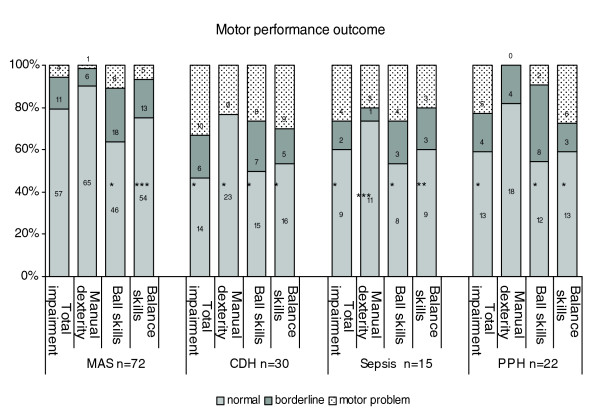
Results of movement assessment battery for all children in each primary diagnosis group. Chi-squared test (observed vs expected distribution): * *P *< 0.001 ** *P *< 0.002 *** *P *< 0.05. One child with pulmonary hypoplasia is not presented in the figure (all scores within the normal range). CDH = congenital diaphragmatic hernia; MAS = meconium aspiration syndrome; PPH = persistent pulmonary hypertension.

Motor performance profiles differed between the primary diagnosis groups, as shown in Figure 2. All test results for the one child with pulmonary hypoplasia were within the normal range (data not presented).

In the group of children with MAS, MABC total impairment scores and manual dexterity scores did not differ from the reference scores, although more problems with ball- and balance skills were found. Also in the PPH group, manual dexterity scores were not deviant. Nevertheless, MABC total impairment scores in this group were deviant on account of problems with ball- and balance skills. In both the CDH and sepsis group, motor performance was significantly lower in all domains. The CDH group scored significantly worse than the MAS group on MABC total impairment score (*Z *= -2.4, *P *< 0.02), balance skills (*Z *= -2.8, *P *< 0.01) and ball skills (*Z *= -2.7, *P *< 0.01), but not on manual dexterity. Differences between the other groups were not significant.

### Intelligence and behavioural scores

One hundred and thirty-one children were assessed with the RAKIT (Figure 1). The IQ scores (mean = 99.7, SD = 18.1, n = 131) and the behavioural scores (mean = 50.2, SD = 9.9; n = 125) did not differ from those for the reference population (*P *> 0.05), neither in the total group nor in the primary diagnosis groups.

### Motor performance relation with health status, cognition, behaviour and socio-economic status

Table 3 shows outcomes for the total group and for the primary diagnosis groups including the children not tested because of already known severe disabilities. Mortality was highest in the CDH group (41.9%) and lowest in the MAS group (6.4%). The CDH group also showed lowest normal outcome in all domains (37.5%) versus 52.6% in the MAS group. Motor domain problems were the problems most frequently encountered in the total group (39.3%), in 22% of all cases combined with cognitive and behavioural problems. Only 12% of the children had cognitive or behavioural problems without motor problems.

**Table 3:** Outcome of neonatal ECMO intervention at the age of five years for the total group and specific diagnosis groups

	Total group n = 224		MAS n = 94		CDH n = 62		Sepsis n = 25		PPH n = 34		Other diagnosis n = 9	
						
	**N**	**%**	**N**	**%**	**N**	**%**	**N**	**%**	**N**	**%**	**N**	**%**
Children died	50	**22.3**	6	**6.4**	26	**41.9**	5	**20.0**	7	**20.6**	6*	**66.6**

Survivors	n = 174 24 missing		n = 88 12 missing		n = 36 4 missing		n = 20 4 missing		n = 27 2 missing		n = 3 2 missing**	

Survivors classified	150	**100**	76#	**100**	32	**100**	16	**100**	25	**100**	1	**100**
Children with severe problems in 2 or 3 domains	20	**13.3**	6	**7.9**	5	**15.6**	4	**25**	5	**20**		
Children with mildly delayed motor development combined with mildly delayed cognitive development and/or behavioural development	13	**8.7**	6	**7.9**	2	**6.2**	2	**12.5**	3	**12**		
Children with mildly delayed motor development and normal cognitive and behavioural development	26	**17.3**	10	**13.2**	11	**34.4**	1	**6.2**	4	**16**		
Children with normal motor development, but mildly delayed cognitive or behavioural development	18	**12**	14	**18.4**	2	**6.2**	2	**12.5**	0	**0**		
Children with normal development in three domains (MABC > p15; IQ > -1 SD; CBCL > p25)	73	**48.7**	40	**52.6**	12	**37.5**	7	**43.8**	13	**52**	1**	100

* diagnosis: 1 Cystic adenomatoid malformation of the lung; 1 pulmonary hypoplasia, 2 pertussis; 1 Idiopathic Pulmonary Hypertension, 1 Acute respiratory distress syndrome

** diagnosis: pulmonary hypoplasia

# inclusive one child not in follow-up with a known syndrome

CBCL = child behaviour checklist; CDH = congenital diaphragmatic hernia; ECMO = extracorporeal membrane oxygenation; IQ = intelligence quotient; MABC = movement assessment battery for children; MAS = meconium aspiration syndrome; PPH = persistent pulmonary hypertension; SD = standard deviation.

None of the six children with cerebral infarction (four with MAS, one with CDH, one with PPH; Table 1) showed normal development. Four had severe problems in all three domains, one child had severe motor problems (hemiparesis) and one child had severe behavioural problems. Of the nine children with cerebral haemorrhage, four scored in the normal domain, three had abnormal development in all three domains, one child had borderline motor problems and one child combined motor and cognitive problems. Six children had clinical insults: four had normal outcome, one had severe cognitive problems and one had severe behavioural problems. Of the 28 children with neonatal seizures shown on electroencephalography (EEG) only nine had normal development, 17 had motor problems (seven severe in more domains, three borderline motor combined with borderline cognitive and/or behavioural problems, seven borderline motor problems) and two had mild cognitive problems.

For the total group no significant relations were found between MABC total impairment scores, socio-economic status or growth outcomes. Negative outcome on the MABC was significantly related to lower IQ-score (*r *= 0.48, *P *< 0.001), and significantly but weakly related to lower behavioural scores (*r *= 0.18, *P *< 0.05), although lower IQ-scores were significantly related to negative behavioural outcome (*r *= 0.32, *P *< 0.001) and SD height (*r *= 0.23, *P *< 0.01).

## Discussion

This study presents five-year outcomes of a nationwide population of 224 neonates treated with VA-ECMO. Severe disabilities in all domains were found in 13.3% of the 174 surviving children. More than half of those with deviant motor performance outcome (26%) had cognitive problems and/or behavioural problems. Children with MAS or PPH had the best outcomes (52% normal). Children with CDH had the worst outcomes (only 37.5% normal in three domains), with more problems in the motor domain, combined with decreased growth. Although not significantly different, cognitive problems were most frequent in the MAS group (18.4%). Perinatal cerebral abnormalities and neonatal seizures as measured by EEG were highly related to deviant outcome.

In this study 24 children (14%) were lost to follow-up. These children did not differ in perinatal or ECMO characteristics, and the percentage of CDH children was somewhat lower and the number of non-native children was higher in the non-responder group. We cannot exclude that outcome will be somewhat worse as a result of disproportionately prevalent poorer outcome in the hard-to-trace subgroups.

The use of standardised tests allowed the comparison of this sample with an age-related reference population. We opted for the MABC because children may have motor performance problems even if the neurological examination is normal [25]. In the MABC assessment protocol children are provoked to perform age-related functional skills as fast and accurately as possible, comparable with the demands in daily life [26], and these demands lace more load on the neurological system.

The literature contains a few earlier, similar studies. ECMO survivors in the UK were tested at ages four and seven years with standardised tests for cognitive and behavioural assessment, with the addition of MABC components at the age of seven years [11,14].

Glass and colleagues [7,13] published two studies focusing on five-year outcome using the same protocols. Controls were recruited from a local paediatric practice. Both studies showed major disability in 17% of the ECMO patients (vs. 13% in our study), motor disability was present in 5 to 6% of the children, and was related to cerebral palsy.

The incidence of severe disability in our study is comparable with that in the study by Glass and colleagues, [7,13] but somewhat higher than in the UK study group (2%). Motor performance problems seem to be more frequent in the UK study group (57 % vs. 33% in our study) [14]. Although similar, the studies are hardly comparable because of different types of control groups, differences in test age, differences in decision rules (means and SD of MABC scores underlined the decisions in the UK study) and selection bias (a selection of MABC test items in the UK study and tasks not related to daily activity in the study by Glass and colleagues). Therefore, we would like to advocate the use of normal-referenced tests to estimate motor performance in the same manner as IQ tests. Another explanation for different findings could be the difference in primary diagnoses in the above mentioned studies.

Functional motor problems interfere with the acquisition of everyday skills and cognitive and social-emotional development in preterm children [27]. In the present study motor problems often went together with cognitive and/or behavioural problems and total MABC scores were significantly related to IQ scores. Although we did not find lower IQ scores on the RAKIT, it should be borne in mind that the predominance of motor morbidity at age five years is likely in part to be due to the relatively young age of the cohort. It is conceivable that increased subtle/cognitive morbidity will become more evident with age, when more academic and cognitive skills are required. Moreover, the short version of the RAKIT focuses on general intelligence and appears relatively insensitive to frontal lobe dysfunction. The UK studies support the hypothesis that motor problems influence the learning of cognitive skills in which movement planning is an important factor: at the age of four years, the ECMO children had specific problems with pattern construction and copying [11], and at the age of seven years had problems with writing [14]. Glass and colleagues also found that the ECMO children had a two-fold risk for academic difficulties at school age and a higher rate of behavioural problems [13].

Bulas and Glass reported that the severity of neonatal brain injury was predictive for neuromotor outcome at five years of age [28]. Still, they also found evidence of compensation following moderate or severe brain injury. A limitation of the present study is the absence of neuro-imaging data in all survivors. However, we found that clinical insults and neonatal seizures on EEG, besides cerebral infarction and haemorrhage, were predictive for adverse outcome. Future research should focus on the precise diagnostics of neonatal injuries and on the presence and influence of therapy programs and/or differences in parental care. These would gain insight into factors improving long-term outcome.

In the present study children born with severe pulmonary hypoplasia or large diaphragmatic defects were at higher risk for motor performance problems. They often also had cognitive and/or behavioural problems and growth scores below -1 SD, indicative of failure to thrive. Studies focusing on long-term morbidity in the CDH population are scarce. Hayward [29] and colleagues found a ventilation/perfusion mismatch and decreased postnatal lung growth in more than half of CDH patients at age one to two years, possibly caused by a limited catch-up growth in the postnatal period. Hamutku [30] and colleagues found lung dysfunction (airway obstruction, hyperinflation and hypoxia at rest) at the age of 9 to 13 years in 50 neonatal ECMO survivors with various primary diagnoses. They could not confirm differences in outcome related to primary diagnosis, possible as a result of the small number of children. Boykin and colleagues found abnormalities in baseline and post-exercise pulmonary functions in 10 to 15-year-old ECMO children with MAS as primary diagnosis [31]. In a multicentre, prospective study it was found that reduction in pulmonary function at eight years was linked to ECMO itself, CDH and small for gestational age [32]. Taken together, it can be concluded that although pulmonary dysfunction seems to be more serious in children with CDH, the ECMO treatment itself also increases risk of pulmonary problems. Future studies are needed into the relations between persistently reduced lung capacity, growth problems and conditional restrictions in motor activities in the ECMO population as a whole and the CDH population in particular.

In the absence of a matched control group it remains difficult to establish the extent to which ECMO treatment itself contributes to the outcome. The UK ECMO trial [1,3,14] did show a benefit for ECMO based on the primary outcome "death or severe disability". In a recent Cochrane review it was established that this is particularly true for infants with no specific problem of lung formation (CDH) [33]. In a recent review focusing on the benefits of ECMO in infants with CDH, Morini and colleagues [34] concluded that ECMO leads to a reduction in mortality. However, in very severe CDH patients this may lead to long-term disability [31,34]. Unfortunately, at this moment most studies concentrate on the first years after ECMO and not on long-term outcome. The scattered data indicate substantial morbidity in long-term survivors of ECMO especially in CDH, including pulmonary damage and neurocognitive defects.

In this study we can confirm that the worst outcome was found in the CDH population: almost 42% of the children died as a neonate, 72% had growth problems, almost 16% had severe disabilities and another 6% had borderline problems in all domains, 34% had borderline motor problems and only 37.5% functioned in the normal domain. Although the outcome for children with MAS was much better, only 53% of the survivors functioned in the normal domain. Although no significant morbidity was seen in children with sepsis, PPH or other diagnosis was also relatively high. In particular children with MAS seem to profit from ECMO intervention and less severe problems are present. The MAS children are the most healthy children in which neurological outcome is determined by an increased risk of perinatal and neonatal hypoxaemia in the first days of life. These children seem to have more diffuse problems in cerebral information processing such as diminished ball and balance skills, and relatively more cognitive and behavioural problems, also reported in children with mild or severe asphyxia during birth.

## Conclusions

This study shows considerable morbidity in ECMO-treated survivors at age five years, which is not greatly different from that reported in previous publications. Decreased motor performance is the most frequent complication, often associated with problems in the cognitive and behavioural domains. The manual dexterity activities are less affected and ball skills and balance skills are most affected. These functional motor problems could interfere with the acquisition of everyday skills, and with later cognitive and social-emotional development. We, therefore, think that longer follow-up of children at risk of morbidity at age five years is required. In particular, the CDH group is at high risk because of failure to thrive. Moreover, perinatal cerebral complications, such as cerebral infarction, cerebral haemorrhage and neonatal seizures at the EEG, were predictive of an adverse outcome. Brain damage and pulmonary dysfunction seem to be important determinants. Precise registration of interventions and long-term outcomes are necessary for scientific evaluation and clinical management of the sequelae and the developmental problems. As local patient groups are usually small, national and international collaboration is recommended. We believe that a successful follow-up programme of the ECMO population should be structured in consultation with representatives from different disciplines such as paediatricians, paediatric physical therapists and psychologists. Improvement of long-term outcome requires not only insight into the primary diagnosis-related factors, the ECMO intervention related factors but also insight into factors stimulating recovery.

## Key messages

• ECMO treatment decreases mortality; however, morbidity is high at age five years: 13% of the children were severely handicapped.

• Only 49% of the ECMO children showed normal outcomes in all domains, and motor performance problems were most often present, often combined with cognitive and behavioural problems.

• Manual dexterity activities are less affected and ball skills and balance skills are most affected.

• Morbidity in primary diagnosis groups differs: high morbidity in CDH children, lower in MAS and PPH children, and relatively high morbidity in the sepsis group.

• Precise registration of interventions and long-term outcomes are necessary for scientific evaluation and clinical management, and strong collaboration between disciplines and centres is required.

## Abbreviations

BMI: body mass index; CBCL: child behaviour checklist; CDH: congenital diaphragmatic hernia; ECMO: extracorporeal membrane oxygenation; EEG: electroencephalography; IQ: intelligence quotient; MABC: movement assessment battery for children; MAS: meconium aspiration syndrome; PPH: persistent pulmonary hypertension; RAKIT: revised Amsterdam intelligence test; SD: standard deviation; VA-ECMO: veno-arterial extracorporeal membrane oxygenation.

## Competing interests

The authors declare that they have no competing interests.

## Authors' contributions

MWGN-vdS participated in the follow-up programme in Nijmegen as a paediatric physiotherapist. She contributed substantial to conception and design of the follow-up programme, participated in data acquisition analysed and interpreted the data and drafted the manuscript. MHMvdC-vZ participated in the follow-up programme in Rotterdam as a paediatric physiotherapist and contributed substantially to data acquisition, analysis and interpretation. AJWM-J participated in the follow-up programme in Nijmegen as a paediatric physiotherapist and contributed substantially to data acquisition, analysis and interpretation. JJCM-R participated in the follow-up programme in Nijmegen as a psychologist and participated in data acquisition, analysis and interpretation of the psychological data. P-M participated in the follow-up programme in Rotterdam as a psychologist and participated in data acquisition, analysis and interpretation of the psychological data. AFJ-vH participated in the perinatal ECMO intervention as a paediatrician and contributed to conception and design of the follow-up programme in Nijmegen and analysis and interpretation of the perinatal dataset. SJ-G participated in the follow-up programme in Rotterdam as a paediatrician and advised in the analysis of the data. D-T and LAA-K participated as medical doctors in the coordination and design of the study in Rotterdam and Nijmegen, respectively; All authors contributed to the draft of the manuscript, read and approved the final manuscript and take responsibility for appropriate portions of the content.
